# Supplementary figures and images for: Evaluation of subretinally delivered Cas9 ribonucleoproteins in murine and porcine animal models highlights key considerations for therapeutic translation of genetic medicines
Source: PLoS One. 2025 Jun 24;20(6):e0317387. doi: 10.1371/journal.pone.0317387 (PMC12186880; doi:10.1371/journal.pone.0317387)

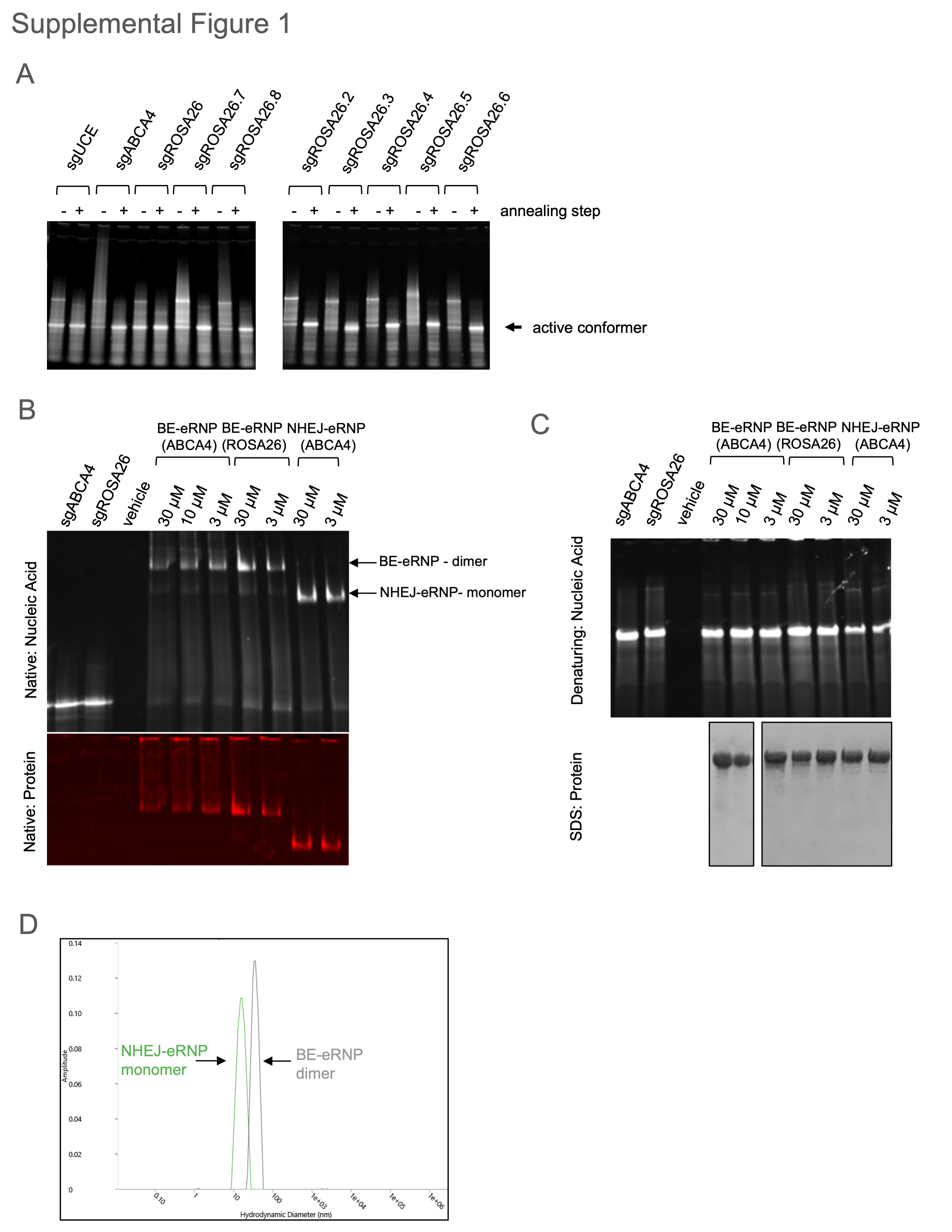

Supplement: S1 Fig — A. Native gel electrophoresis of sgRNAs prior to eRNP assembly to evaluate RNA conformation. B. Native gel electrophoresis of assembled NHEJ-eRNPs and BE-eRNPs to determine quaternary structure. C. Denaturing PAGE and SDS-PAGE of NHEJ-eRNPs and BE-eRNPs to analyze purity of total RNA and protein. D. Dynamic light scattering of NHEJ-eRNP and BE-eRNP. gRNA annotations are described in S2 Table. (TIFF) [file pone.0317387.s001.tiff]

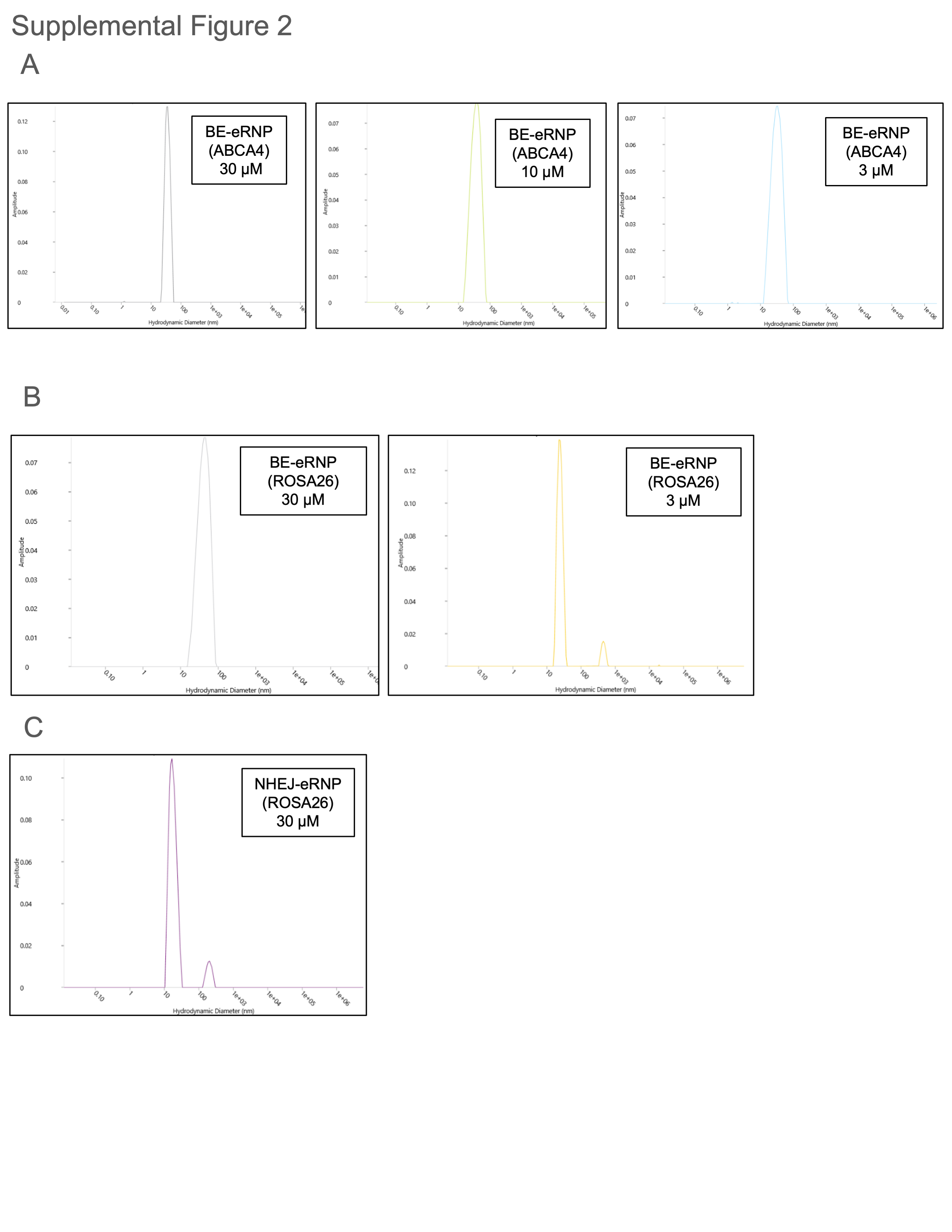

Supplement: S2 Fig — A. BE-eRNP (assembled with sgRNA targeting Abca4 locus) at 3 different dosing concentrations. B. BE-eRNP (assembled with sgRNA targeting the Rosa26 locus) at 2 different dosing concentrations. C. NHEJ-eRNP (assembled with sgRNA targeting the Rosa26 locus) at a single dosing concentration. (TIFF) [file pone.0317387.s002.tiff]

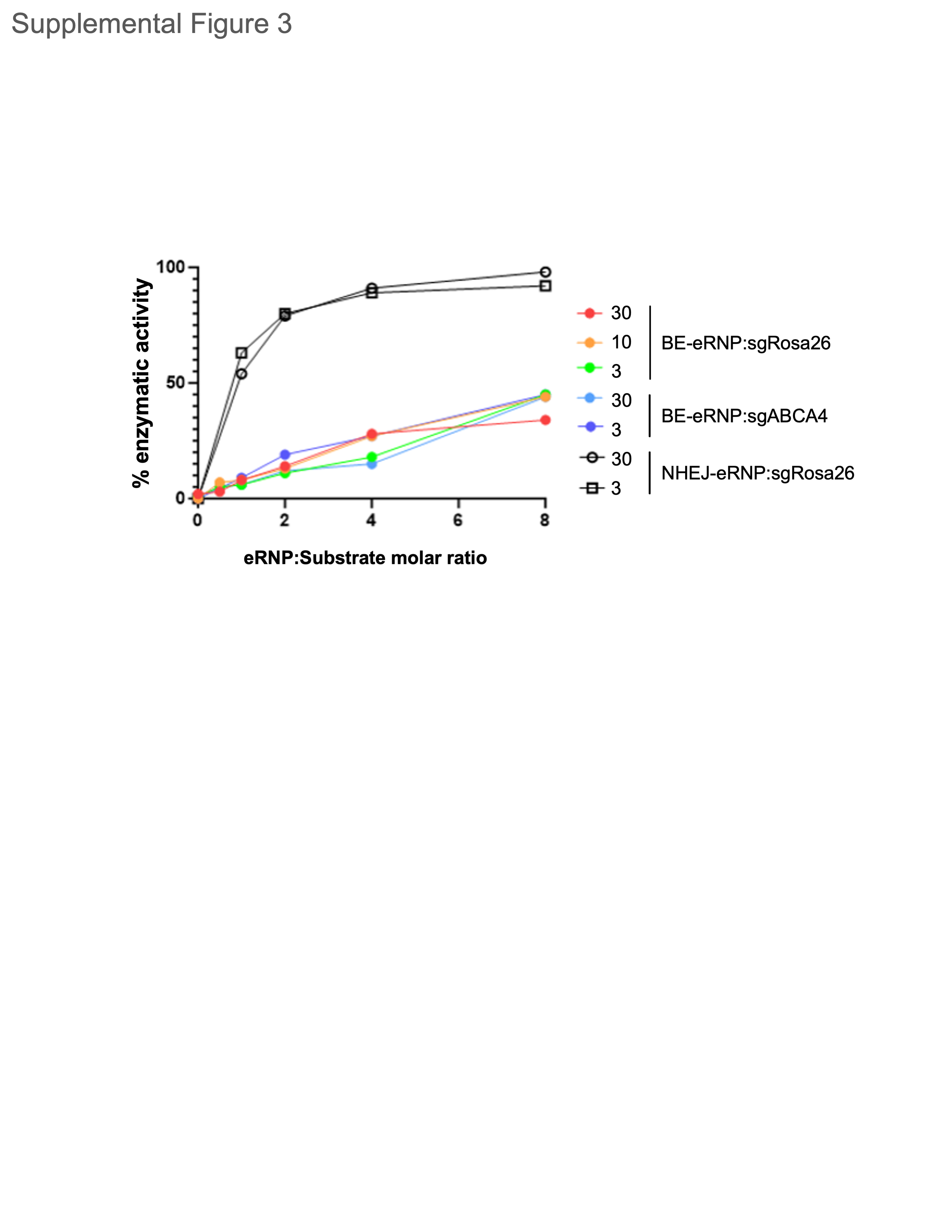

Supplement: S3 Fig — In vitro DNA cleavage (NHEJ-eRNPs) and adenosine deamination (BE-eRNPs) assay data are displayed. Each eRNP sample provided for the animal study shown in main text Figs. 3 and 4 was diluted to the same set of final concentrations for the assay. The eRNP samples are identified in the legend as their dose and contents. The enzymatic activity is shown as the percent of substrate modified as calculated for samples’ respective assay formats (see Methods). (TIFF) [file pone.0317387.s003.tiff]

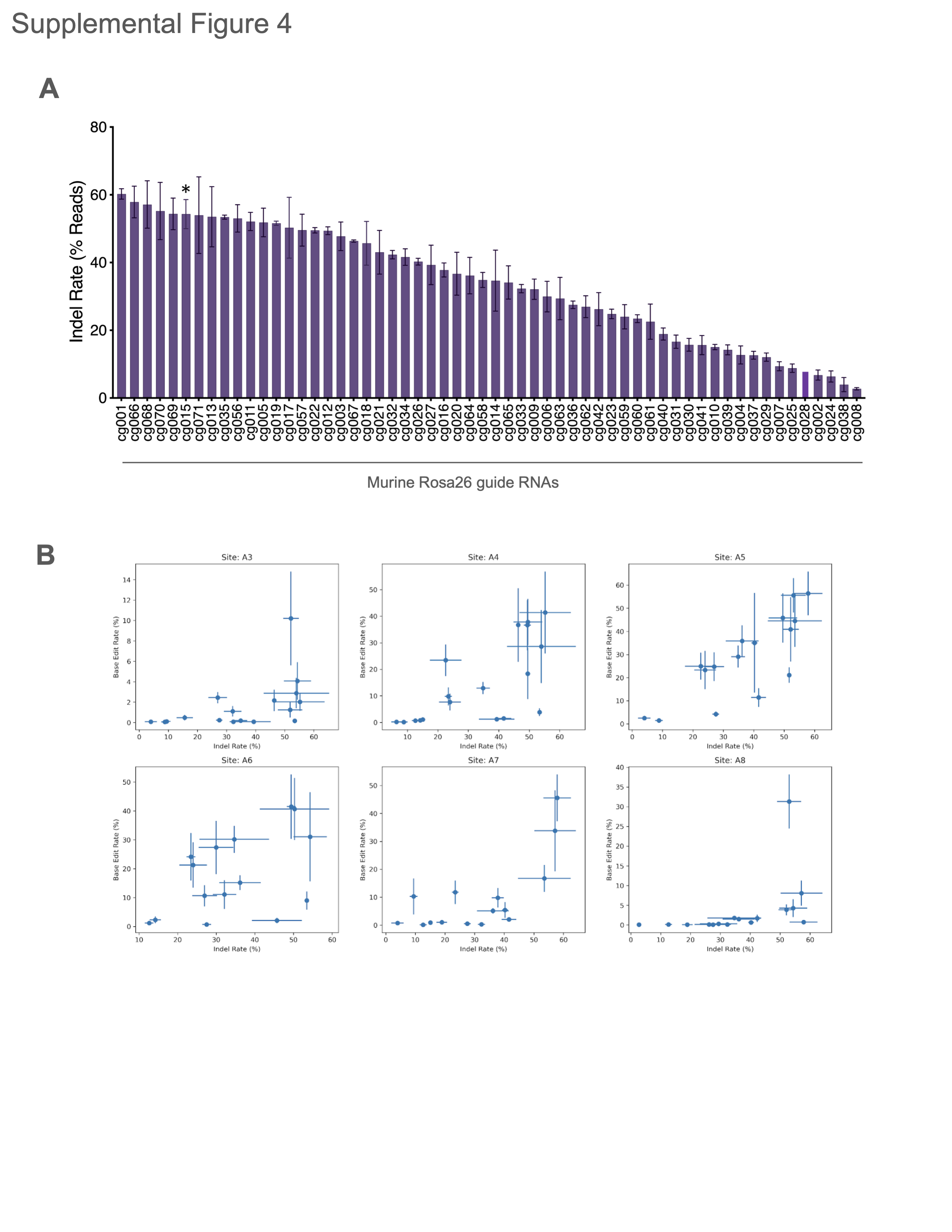

Supplement: S4 Fig — A. Mouse T cell editing rates versus gRNA for NHEJ-eRNPs. NHEJ-eRNPs were complexed with various gRNAs targeting the Rosa26 locus and then used to treat stimulated mouse T cells. NHEJ-eRNPs were complexed with the indicated gRNAs in crRNA:trcrRNA format and then nucleofected into cells. Editing rates were quantified based on the frequency of reads containing indels from Illumina sequencing. The asterisk indicates the crRNA corresponding to the targeting gRNA used in main text Fig. 2B and 2C. B. Frequency of A → G transitions upon ABE-eRNP treatment vs. frequency of indels upon NHEJ-ABE treatment at each A position across protospacers tested in Fig. 2A and panel A of this figure. For the A position indicated in each panel, the ABE and indel rates for subset of gRNAs with an A at that position were plotted against each other. Each point corresponds to the mean editing rate for one gRNA and error bars represent the standard deviation across biological replicates. (TIFF) [file pone.0317387.s004.tiff]

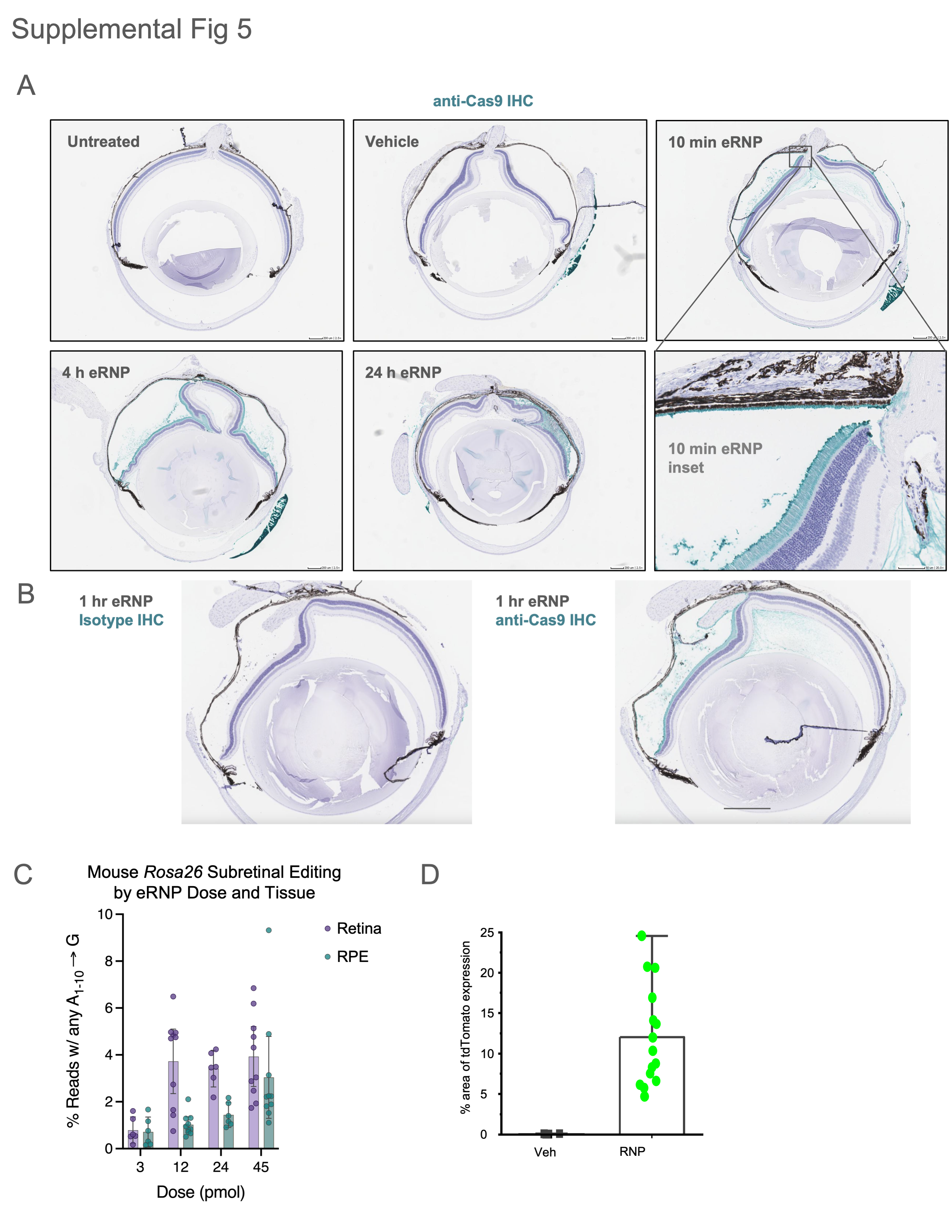

Supplement: S5 Fig — A. Representative images of murine eyes treated with subretinal administration of 2 µl of vehicle or eRNP at denoted time points with an untreated eye control. Sections were stained by anti-Cas9 immunohistochemistry (teal). The outer nuclear layer is denoted with a black arrow and the RPE is denoted with a blue arrow. B. Representative images of murine eyes 1 hour post subretinal administration with 1 µl eRNP stained with isotype control (left) or anti-Cas9 immunohistochemistry (right, teal) C. Editing rates by tissue following subretinal injection of Rosa26-targeting ABE-eRNPs complexed with mmRosa_sg1 in mice at four doses. Five days following subretinal administration, mice were sacrificed and their eyes dissected to separate the neural retina from the eyecup, and then RPE layer was separated from the choroid. Editing rates from Illumina DNA amplicon sequencing are shown as mean ± SD across eyes at each dose for retina and RPE tissue samples. Each overlaid point corresponds to a single eye. Reads were scored as positive for editing if at least one A → G transition was detected within a 10 base edit window. These data were collected with the same sample shown in Fig. 3 at a different study site and with a different operator. (TIFF) [file pone.0317387.s005.tiff]

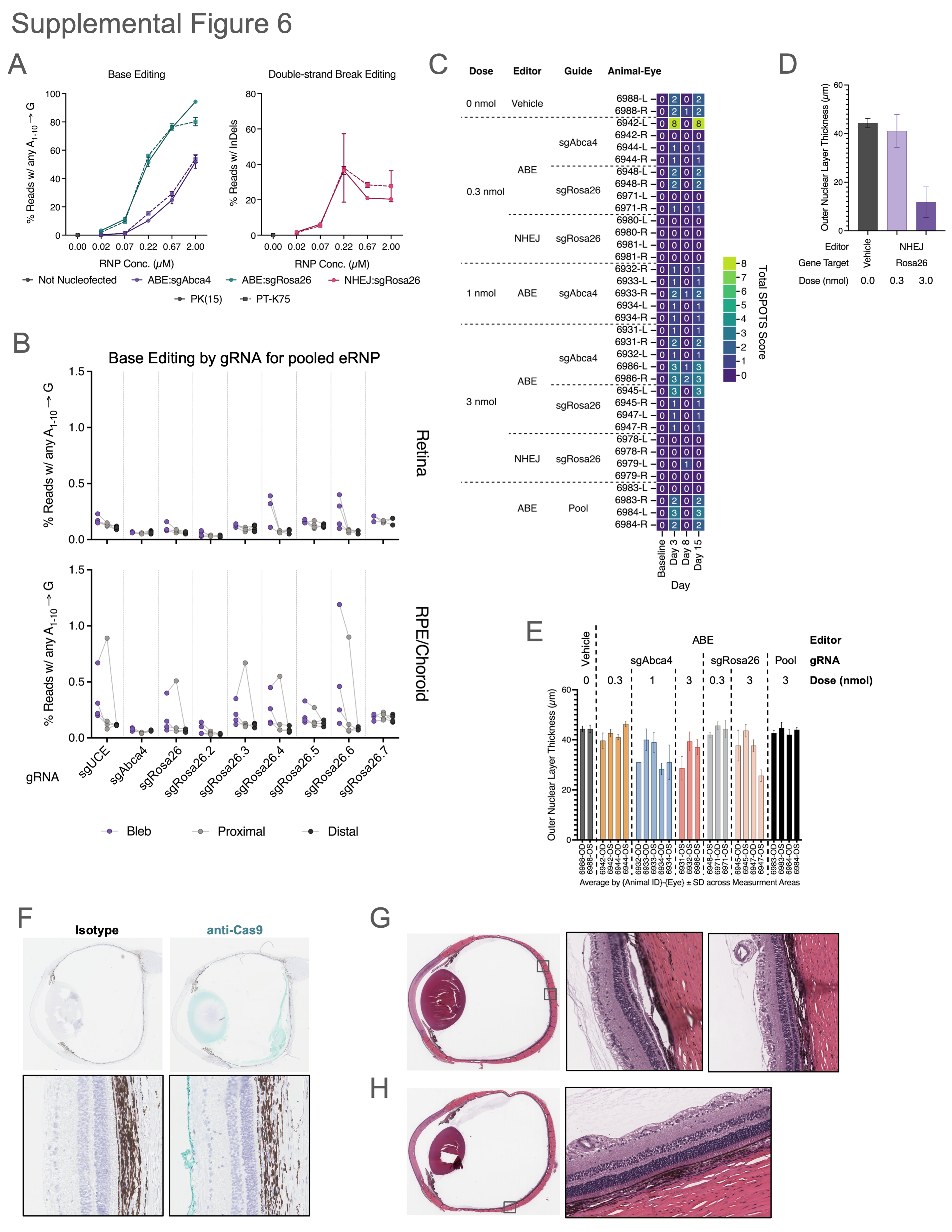

Supplement: S6 Fig — A. Adenine base editing and indel formation following nucleofection of BE-eRNPs (left panel) and NHEJ-eRNPs (right panel) in porcine cell lines PK [15] and PT-K75 at various concentrations with two different gRNAs. Editing rates calculated from Illumina DNA amplicon sequencing are plotted as mean ± standard deviation from three biological replicates. The indicated RNP concentrations correspond to the final mixture with cells in the nucleofection cuvette. B. Editing rates as shown in (A) and (B) for eyes treated with a pool of ABE-eRNPs each complexed with a different gRNA. Editing rates corresponding to the intended gene target sequence are shown for each gRNA and arranged by tissue. C. Aggregate SPOTS scores per minipig porcine eye following subretinal administration of eRNP. D. Outer nuclear layer thickness measured by OCT plotted for NHEJ-eRNP groups. Data are plotted as the mean ± standard deviation for each group across all measurements in all eyes. E. Outer nuclear layer thickness measured by OCT plotted by eye and grouped by eRNP, gRNA, and dose. Data are plotted as the mean ± SD across measurement areas (regularly spaced 2D OCT slices spanning the superior and inferior regions proximal to the superior injection bleb) collected for each eye. F. Representative images of porcine eyes 2 weeks post subretinal administration stained with isotype control or anti-Cas9 immunohistochemistry (teal). Representative whole eye scans and high magnification (inset) images of H&E-stained porcine eyes 2 weeks post subretinal administration with G) 3 nmol or H) 3 nmol NHEJ eRNP. (TIFF) [file pone.0317387.s006.tiff]

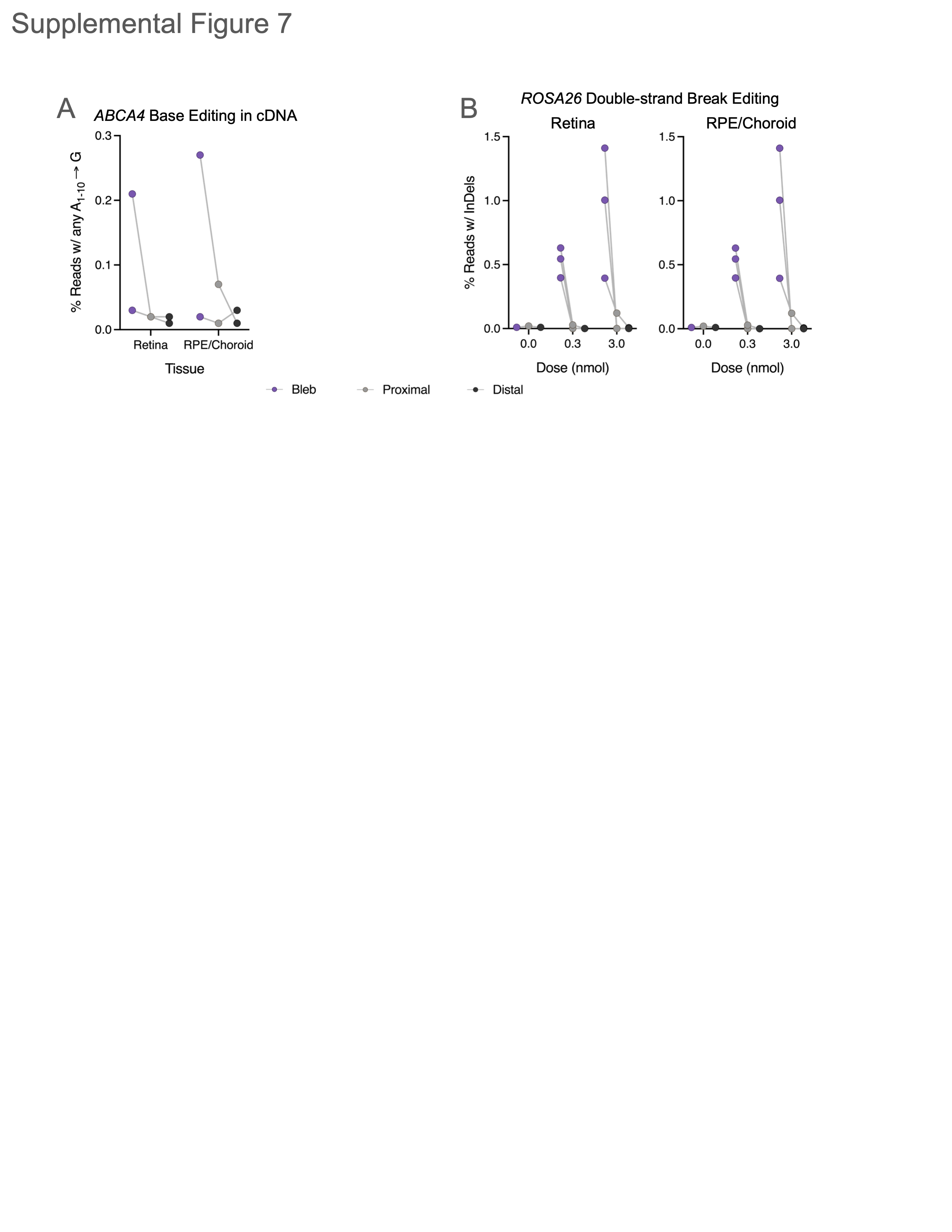

Supplement: S7 Fig — A. Editing rates by tissue following subretinal injection of ABCA4-targeting ABE-eRNPs complexed with sgABCA4 in minipigs. Two weeks following subretinal administration, animals were sacrificed and eyes were dissected to collect intact tissue layers for the neural retina and the choroid + retinal pigmented epithelium. For each tissue, three biopsy punches were taken corresponding to the injection bleb, tissue immediately adjacent to the bleb, and a region distal to the bleb. Editing rates observed in reverse-transcribed cDNA from each sample from Illumina amplicon sequencing are plotted for each eye by tissue and dose as described in Fig. 4. B. Editing rates by tissue following subretinal injection of ROSA26-targeting NHEJ-eRNPs complexed with sgRosa26 in minipigs at multiple doses. Editing rates from Illumina DNA amplicon sequencing are plotted for each eye by tissue, biopsy region, and dose. Reads were scored as positive if they had an InDel at the expected cut site. (TIFF) [file pone.0317387.s007.tiff]

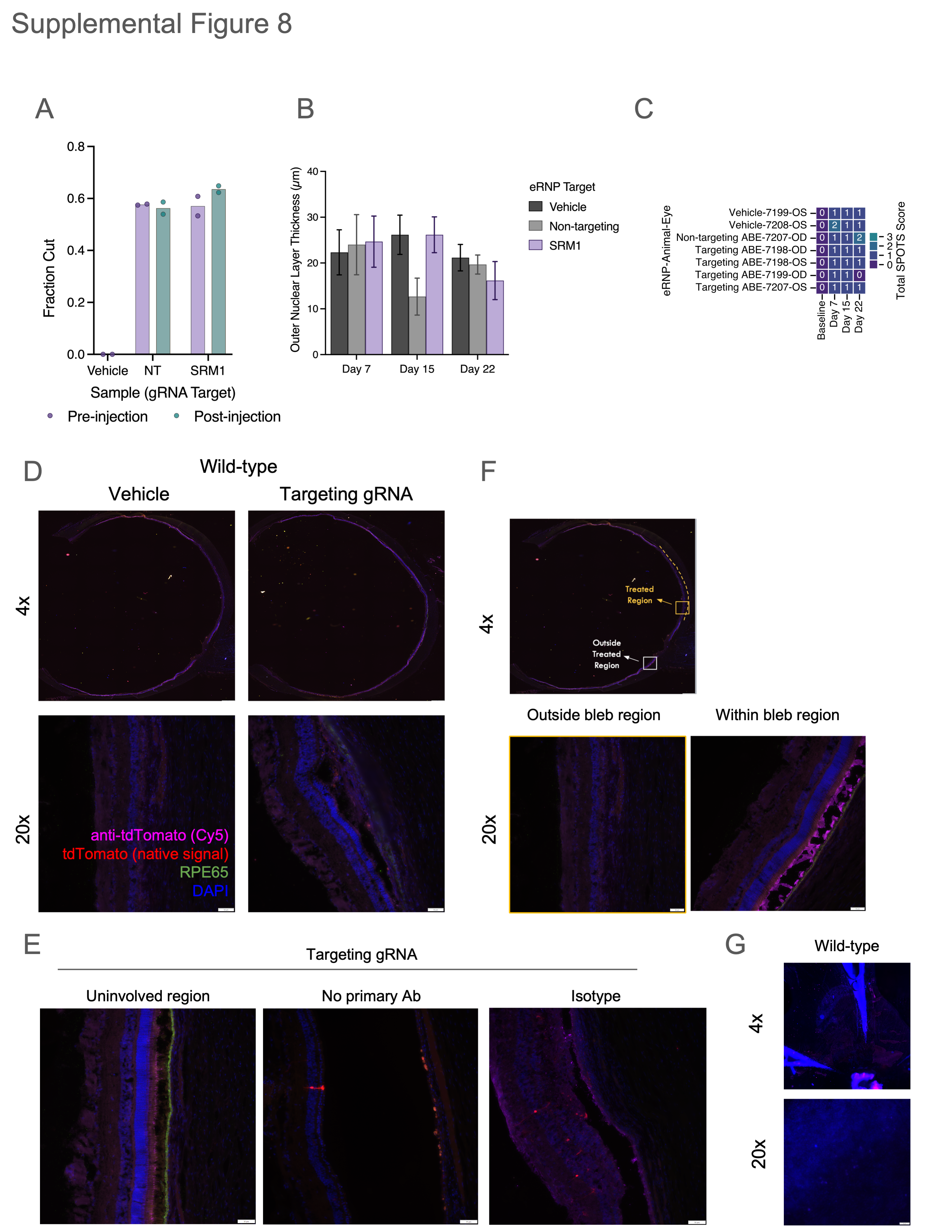

Supplement: S8 Fig — A. Plasmid cutting by purified NHEJ-eRNP samples complexed with non-targeting (NT) and reporter locus gRNAs (SRM1). Sample aliquots were collected and stored before shipment to the test site (pre-injection) and from the material recovered from the injector following administration (post-injection), and then assayed side-by-side. Each point corresponds to a separate reaction. B. Outer nuclear layer thickness according to OCT at different time points post injection following NHEJ-eRNP administration in the SRM1 pig study described in Fig. 5. Data are plotted as the mean ± standard deviation for each group across all measurement areas from superior (injected) regions. C. Aggregate SPOTS scores per porcine eye following subretinal administration of eRNP. D. Fluorescent images of a wild-type littermate (from SRM1 strain) porcine eyes treated with either vehicle or targeting gRNA NHEJ-eRNP. E. Fluorescent images from a SRM1 porcine eye from either the uninvolved inferior region, stained without anti-tdTomato primary antibody, or with isotype antibody (corresponding to the anti-tdTomato antibody). F. Fluorescent image at 4× and 20 × magnification within the treated (superior) and uninvolved (inferior) region of a SRM1 porcine eye. G. Fluorescent images of a flat-mounted RPE/Choroid/Sclera from a wild-type littermate control (from SRM1 colony) eye at 4× and 20 × magnification. (TIFF) [file pone.0317387.s008.tiff]
