# Supplementary material for: Evaluation of subretinally delivered Cas9 ribonucleoproteins in murine and porcine animal models highlights key considerations for therapeutic translation of genetic medicines
Source: PLoS One. 2025 Jun 24;20(6):e0317387. doi: 10.1371/journal.pone.0317387 (PMC12186880; doi:10.1371/journal.pone.0317387)
Supplement: S1_raw_images — Raw images (PDF) [file pone.0317387.s012.pdf]

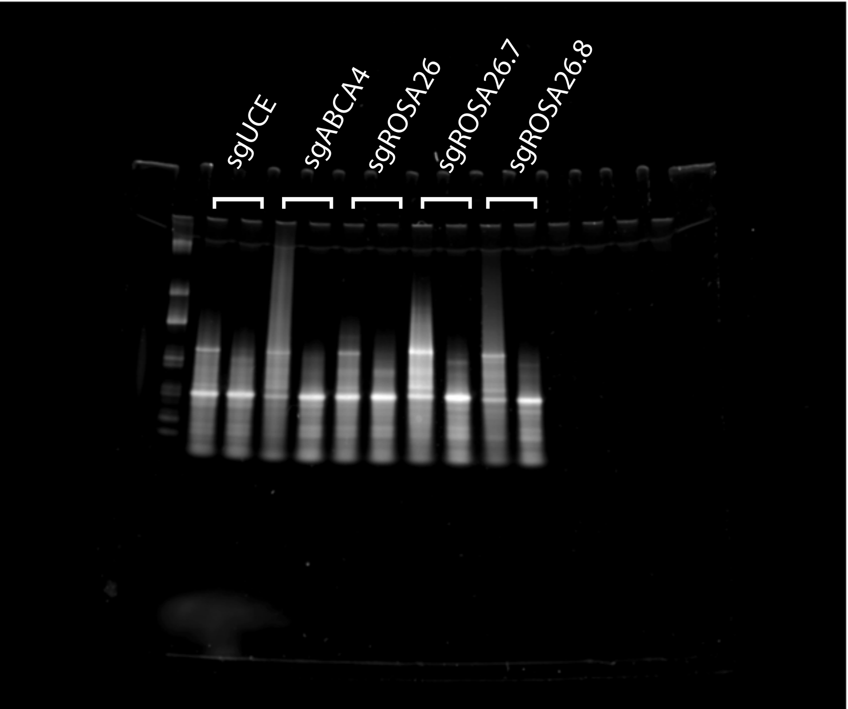
sgUCE
sgABCA4
sgROSA26
sgROSA26.7
sgROSA26.8

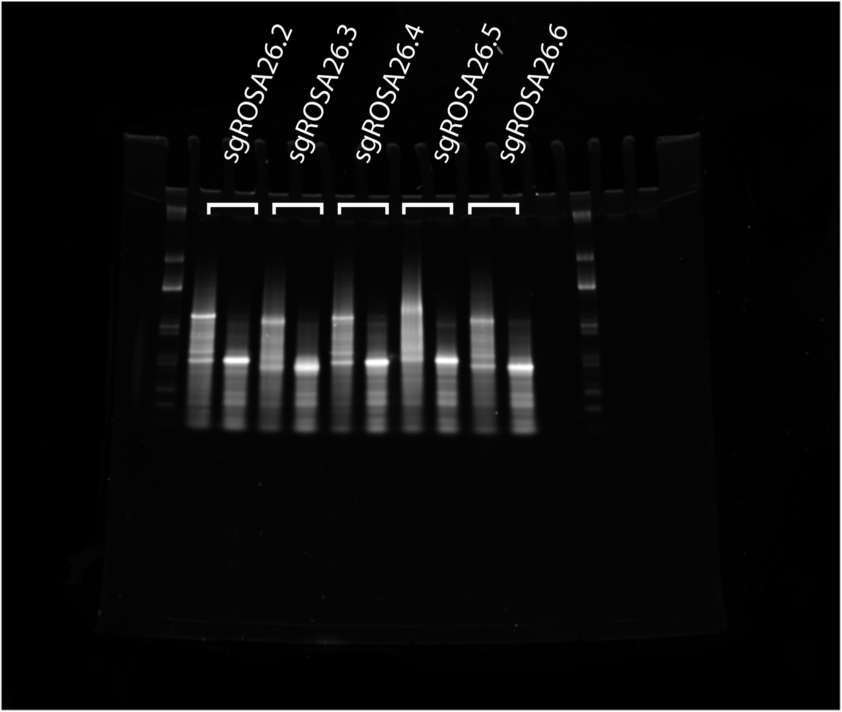

sgROSA26.2
sgROSA26.3
sgROSA26.4
sgROSA26.5
sgROSA26.6

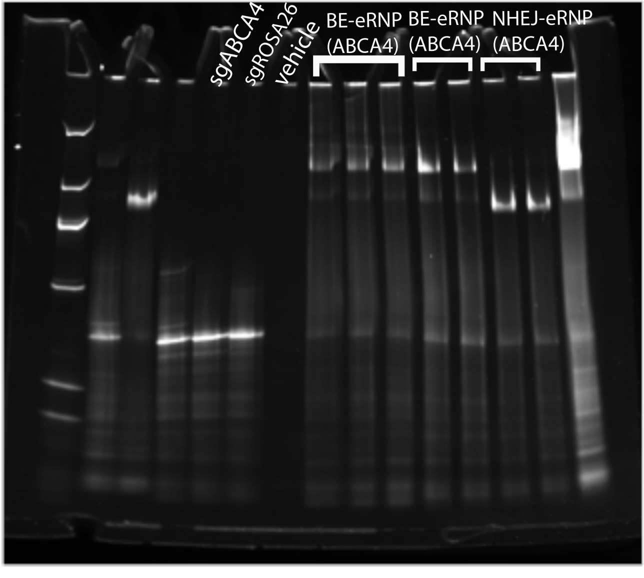
sgABCA4
sgROSA26
vehicle
BE-eRNP
(ABCA4)
BE-eRNP
(ABCA4)
NHEJ-eRNP
(ABCA4)

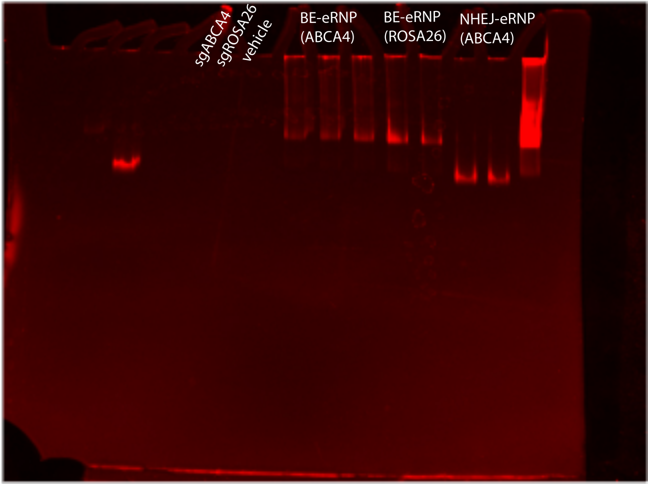
sgABCA4
sgROSA26
vehicle
BE-eRNP
(ABCA4)
BE-eRNP
(ROSA26)
NHEJ-eRNP
(ABCA4)

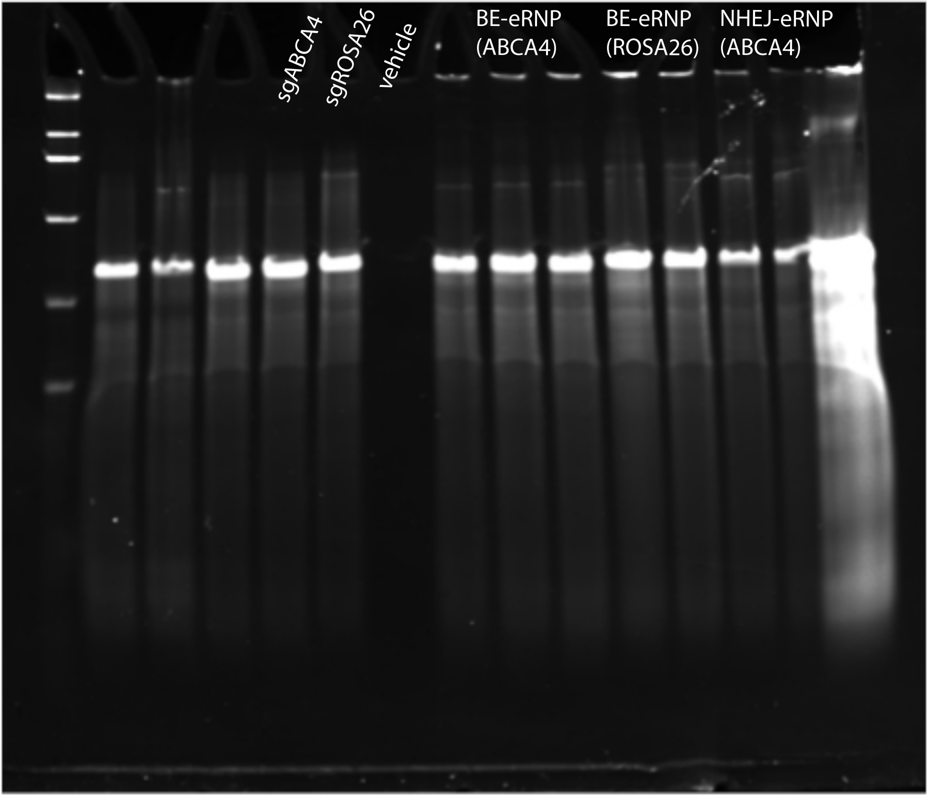
sgABCA4
sgROSA26
vehicle
BE-eRNP
(ABCA4)
BE-eRNP
(ROSA26)
NHEJ-eRNP
(ABCA4)

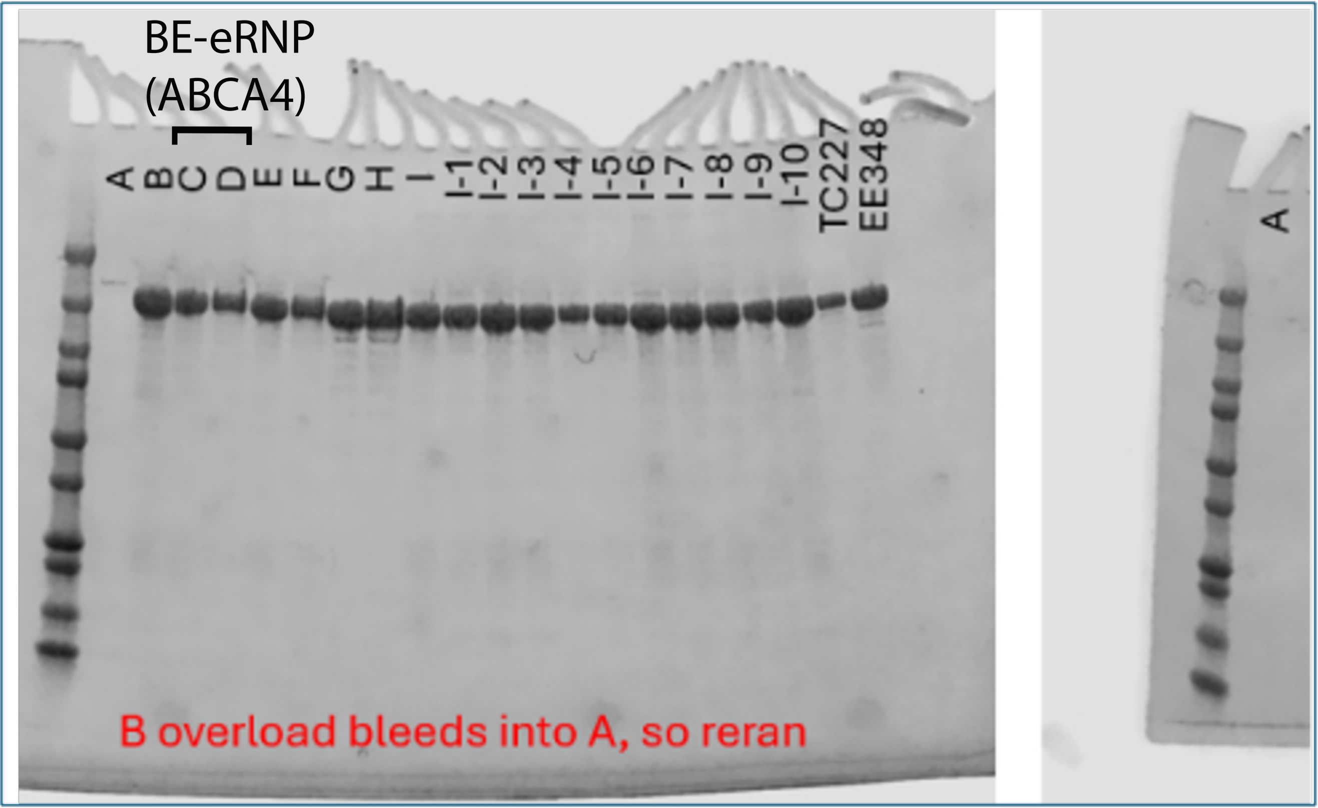
BE-eRNP
(ABCA4)
A
B
C
D
E
F
G
H
I
I-1
I-2
I-3
I-4
I-5
I-6
I-7
I-8
I-9
I-10
TC227
EE348
B overload bleeds into A, so reran
A

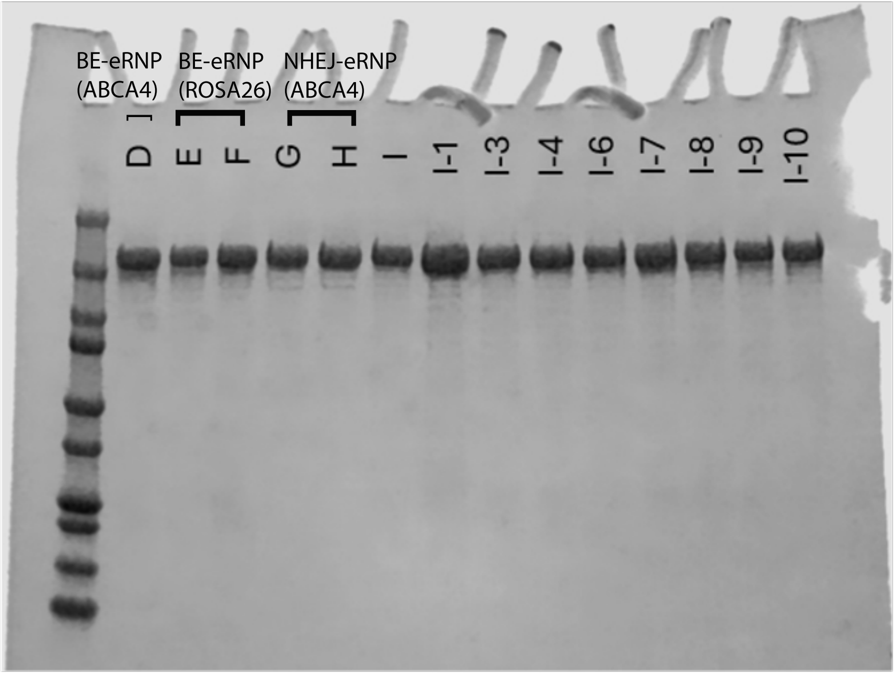
BE-eRNP
(ABCA4)
BE-eRNP
(ROSA26)
NHEJ-eRNP
(ABCA4)
D
E
F
G
H
I
I-1
I-3
I-4
I-6
I-7
I-8
I-9
I-10
